# Rib cartilage grafting in upper limb surgery: an overview

**DOI:** 10.1051/sicotj/2015003

**Published:** 2015-06-19

**Authors:** Laurent Obert, François Loisel, Florelle Gindraux, Yves Tropet, Daniel Lepage

**Affiliations:** 1 Orthopaedic and Traumatology Surgery Service, University Hospital of Besançon 25000 Besançon France; 2 Intervention, Innovation, Imagery, Engineering in Health (EA 4268), Medical and Pharmacology Section, IFR 133, University of Franche-Comté 25000 Besançon France; 3 Clinical Investigation Center in Biotherapy, University Hospital of Besançon France

**Keywords:** Chondrocostal graft, Osteoarthritis, Upper limb surgery, Rib cartilage

## Abstract

*Introduction*: Used routinely in maxillofacial reconstructive surgery, the chondrocostal graft is also used in hand surgery. The purpose of this overview was to analyze at long follow-up the radiological and histological evolution of this autograft, in the hand and wrist surgery.

*Materials and methods*: Since 1992, 144 patients have benefitted from a chondrocostal autograft: 116 osteoarthritis of the thumb carpometacarpal joint, 18 radioscaphoid arthritis, six articular malunions of the distal radius, four kienbock, and four traumatic loss of cartilage of the PIP joint. Magnetic Resonance Imaging (MRI) was performed in 19 patients and histological study in 12 patients with a mean follow-up of 68 months (4–159).

*Results*: Whatever the indication, the reconstruction by a chondrocostal or ostochondrocostal graft has allowed us to obtain satisfactory clinical results at long follow-up. The main question was the viability of the graft. The radiological study has shown the non-wear of the graft and a certain degree of ossification. The MRI confirmed a very small degree of osseous metaplasia but its viability. The biopsies showed a neo-vascularization of the cartilage.

*Conclusion*: Despite the strong mechanical strain in the hand and wrist, the chondrocostal graft is a biological arthroplasty, trustworthy and secure at long time even if it can cause infrequent complications inherent to this type of surgery. Despite the inevitable histological modification, the cartilage remains alive and is of satisfactory quality at long term follow-up and fulfilling the requirements for interposition and reconstruction of an articular surface.

## 1 Introduction

Rib cartilage harvested from the osteocartilaginous junction of the 5th or 6th rib is one component of the traditional armamentarium in maxillofacial surgery for reconstructing the concha [3], mandibular condyle in adults and children [19, 20], nose, and trachea [24]. More recently, orthopedic surgeons have used cartilage grafts, harvested from the ribs or other sites, in post-traumatic proximal interphalangeal (PIP) [12] and metacarpophalangeal (MCP) [29] joint reconstructions for treating finger [15, 33] and radioscaphoid osteoarthritis [17, 31]. Osteochondral allografts and cartilage autografts are now used to address osteochondritis at the elbow and knee [1, 5, 9, 32]. Although cartilage grafts can be harvested from various sites (auricle, nasal septum, rib cartilage), these different types of cartilage are histologically similar [8, 11]. But at this point, we do not know how well a rib cartilage will hold up over time when placed in a limb that is subjected to loading.

## 2 Historical perspective: histological changes

### 2.1 Cartilage and perichondria studies: from animals to humans

**Figure 1.**
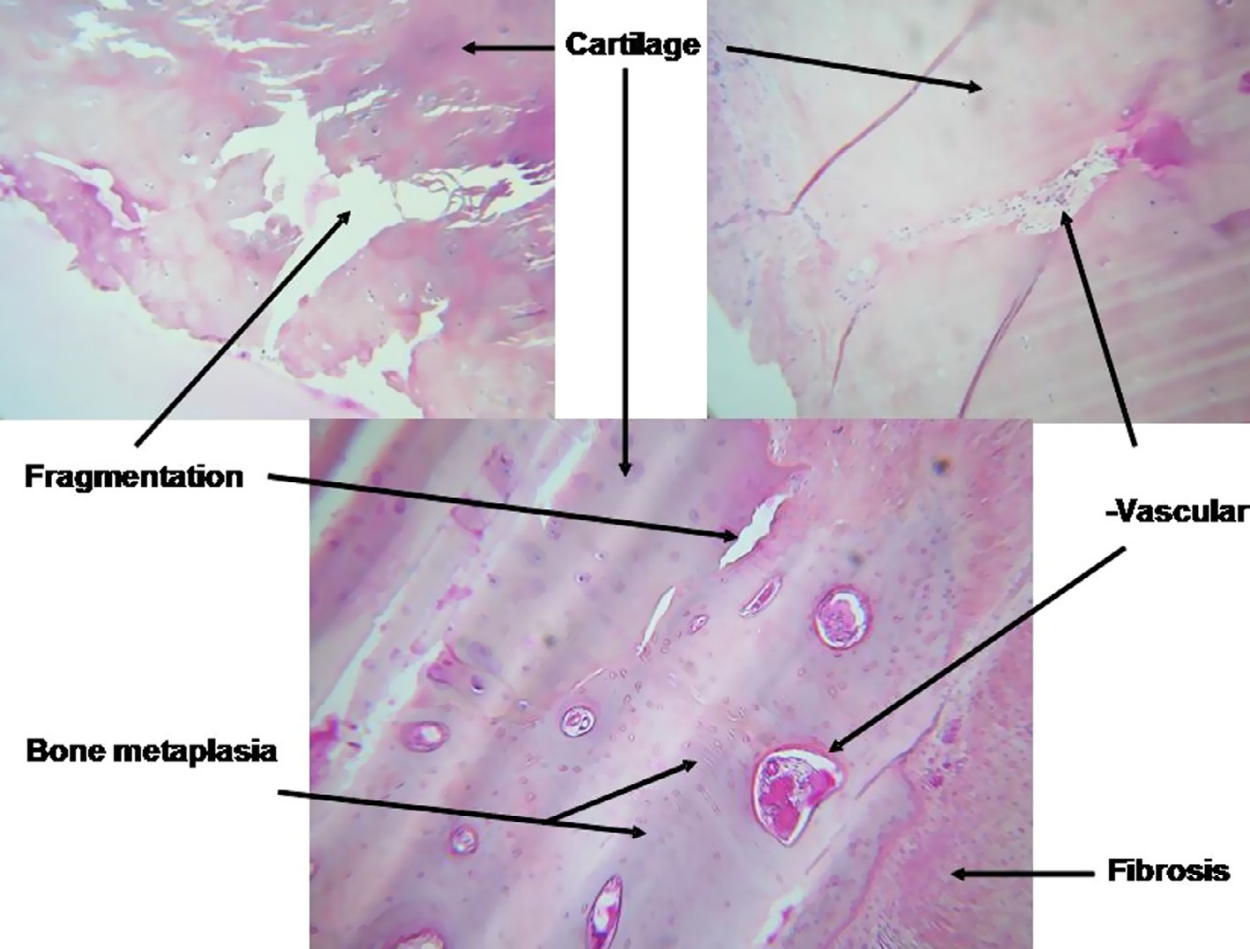
Histological analysis of explanted costal cartilage.

In 1869, Peyraud [27] evaluated the properties of the perichondrium in the dog, compared it to the periosteum, and proposed several hypotheses about the properties of costochondral grafts, all of which were verified over time. Borst [2] arrived at the same conclusion as to the importance of the perichondrium in the survival of free cartilage grafts. In 1984, Duncan et al. [7] evaluated the survival of a free cartilage fragment in an ectopic (subcutaneous) location: cartilage fragments with intact perichondrium had better mechanical properties. Other authors [18, 23] also observed that neoformation of cartilage originated in the perichondrium. Upton et al. [37] and later Gubisch et al. [11] demonstrated the presence of neovascularization within cartilaginous areas transplanted in the rabbit. Svensson et al. [34] studied the role of vascularization in graft survival and graft integration. On the bone side, vascularization originates in the recipient bone; on the cartilage side, it comes from the soft tissue and the environment via imbibition. The kinetics of revascularization are more rapid on the cartilaginous side [34]. However, no newly formed blood vessels have been found between bone and cartilage. Finally, Davis [6], Brunner [4], and Gubisch et al. [11] have shown that the perichondrium plays an important role in protecting cartilage against the fibroblastic invasion responsible for chondrocyte destruction. Transplanted cartilage seems to be subjected to a number of microscopic and structural modifications. Cartilage neovascularization and the presence of perichondrium are the main players in these adaptations. However, the cartilage itself has been shown to survive. Krüger [16] found 48–85% living chondrocytes in seven rib cartilage grafts implanted up to 40 years previously. Ortiz-Monasterio et al. [25] found no differences in terms of graft quality for reconstructing the nasal wall between various graft harvest sites (nasal, auricular, or rib cartilage). Other than a graft’s properties, two phenomena can limit its use, no matter where its source: graft resorption and osseous metaplasia of the cartilage (Figure 1).

### 2.2 Graft resorption

Ortiz-Monasterio et al. [25] observed only 13 partial resorptions out of the 674 cartilage implantations. Brent [3] observed only 1.5% reduction in graft size out of 270 auricular reconstructions using rib cartilage at 5.3 years of follow-up. In the face area, cartilage grafts are stable over time. In the fingers, Hasegawa and Yamano [12] reported two cases of rapidly expanding chondrolysis in patients who underwent PIP joint reconstruction. Interestingly, these were the only two patients who had received pure rib cartilage grafts instead of osteochondral grafts. The author suggested that the cartilage grafts had failed to integrate with the subchondral bone. However, their statements were based on radiological findings and not histological ones. Furthermore, the possibility of sepsis cannot be ignored as the study involved patients with gunshot wounds.

### 2.3 Osseous metaplasia

Osseous metaplasia can be observed on the cartilage side of the graft, even on plain X-rays. The rib cartilage contains hyalin cartilage, which has the characteristics of an epiphyseal plate [28, 30]. Skoog and Johansson [33] reported using costal perichondrium to resurface areas damaged by osteoarthritis in the MCP joint. The perichondrium showed a regenerating effect on cartilage; when the cartilage was in contact with vascularized bone, it tended to undergo osseous metaplasia. Peltomäki et al. [26] reported on seven rib osteochondral grafts that had been explanted because of growth impairment in mandibular reconstructions in children. They systematically found typical ossification zones or zones with osseous metaplasia of the cartilage stemming from chondrocyte transformation.

### 2.4 Relationship between perichondrium, regeneration, osseous metaplasia, and cartilage neovascularization

**Figure 2.**
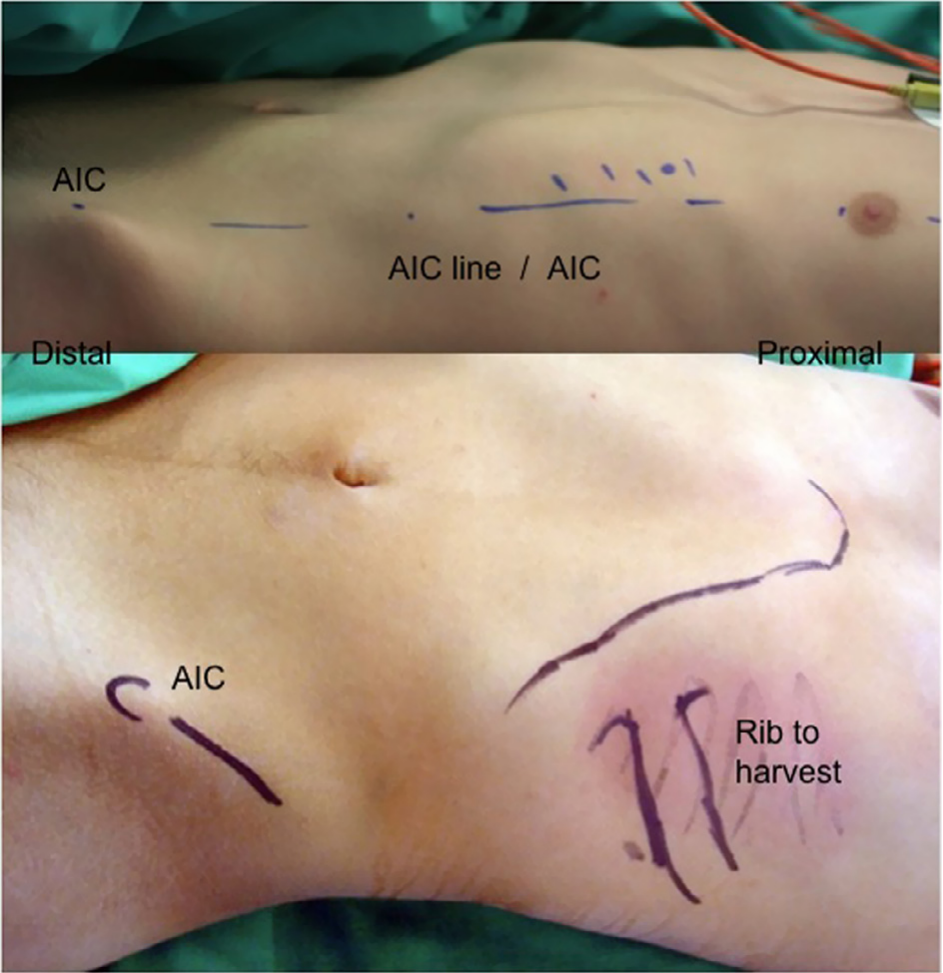
Landmarks of harvesting the graft: the junction between bone and cartilage is located approximately on the line joining mamelon and iliac crest (AIC).

Tropet et al. [36] histologically analyzed 10 free osteochondral grafts. Hyalin cartilage was found in every case, but there were several morphological, structural, and architectural changes. First, the cartilage had fragmented (harvest at two months). As in basic science experiments [7, 34, 37], the neovascularization responsible for fragmentation had penetrated the graft. Chondrocytes were replaced by a fibrous substance. Osseous metaplasia progressively appeared around fibrovascular buds, characterized by chondrocytes with individual focal calcifications. The latter were transformed into bone cells and progressively organized themselves into true Haversian systems, mixed with cartilage remnants and fibrous tissue. Greater amounts of the original cartilage were found as one moved away from these vascularized areas in the graft (Figure 2).

## 3 Rib graft harvesting (Figures 2, 3)

Through a horizontal incision, the anterior cartilaginous end of the 7th, 8th, or 9th rib is exposed. The osteochondral junction is easy to identify because of its different color. The deep side of the 9th rib is reamed carefully. The graft is harvested using a no. 23 scalpel blade. The rib harvest is extraperichondrial for greater safety. The perichondrium can also be harvested. If the rib is sufficiently wide, the graft can be harvested without breaking rib continuity. In collaboration with the anesthesiologist, positive pressure insufflation allows the surgeon to verify pleural integrity. After closing the intercostal muscles and subcutaneous tissues, a subcuticular suture is used to close the wound with placement of a Redon drain.

**Figure 3.**
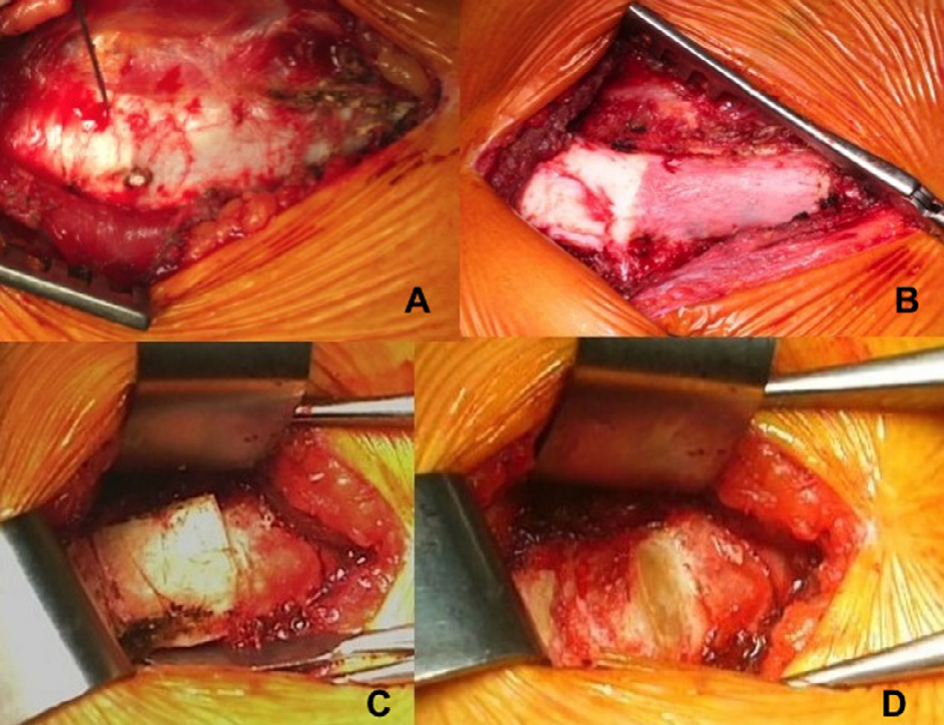
The osteochondral junction is easy to identify: after anterior surgical approach of the 8th rib (A), cartilage is white and soft and can be resected easely, the osseous part is pink (B) and has to be cut with a saw (C). Surgical site after ablation of the graft (D).

## 4 Radiological appearance of rib cartilage

Tropet et al. [36] performed an MRI of rib cartilage in a healthy volunteer and showed that this cartilage had the same signal as hand cartilage: an isointense signal on T1-weighted images and a hypointense signal on T2-weighted images. On CT images, the graft had a slight hyperintensity. However, the signal intensity depends on the constants used for the imaging exam and cannot be used to analyze a graft’s structure. There were not enough images to establish any rules for reading radiological evaluations of rib cartilage, but they served as a reference for possible structural modifications of the graft in the hand over time.

## 5 Uses in the upper limb

### 5.1 Treatment of thumb basal joint (CMC) arthritis (Figure 4)

Basal joint arthritis causes pain and reduced key pinch ability in patients who are still active [35, 36]. Trapeziectomy is the gold-standard treatment. Randomized series have not demonstrated any additional benefit of ligament reconstruction. Despite disadvantages related to CMC joint implants such as dislocation, loosening, wear, and infection, some authors consider them to be the best solution because they quickly provide the patient with better function. Although this is a credible argument, it remains to be proven. Tropet et al. [36] introduced cartilage grafting as a treatment for basal joint arthritis more than 15 years ago. This technique incorporates partial trapeziectomy. The rib graft is harvested at the osteochondral junction; the graft is remodeled by a scalpel (no. 23), corresponding to the dimensions of the cavity and the cartilaginous portion of the graft is placed in contact with the metacarpal base, the bone part opposite the trapezium remaining. As a result, this procedure is a partial trapeziectomy combined with a free osteochondral graft.

**Figure 4.**
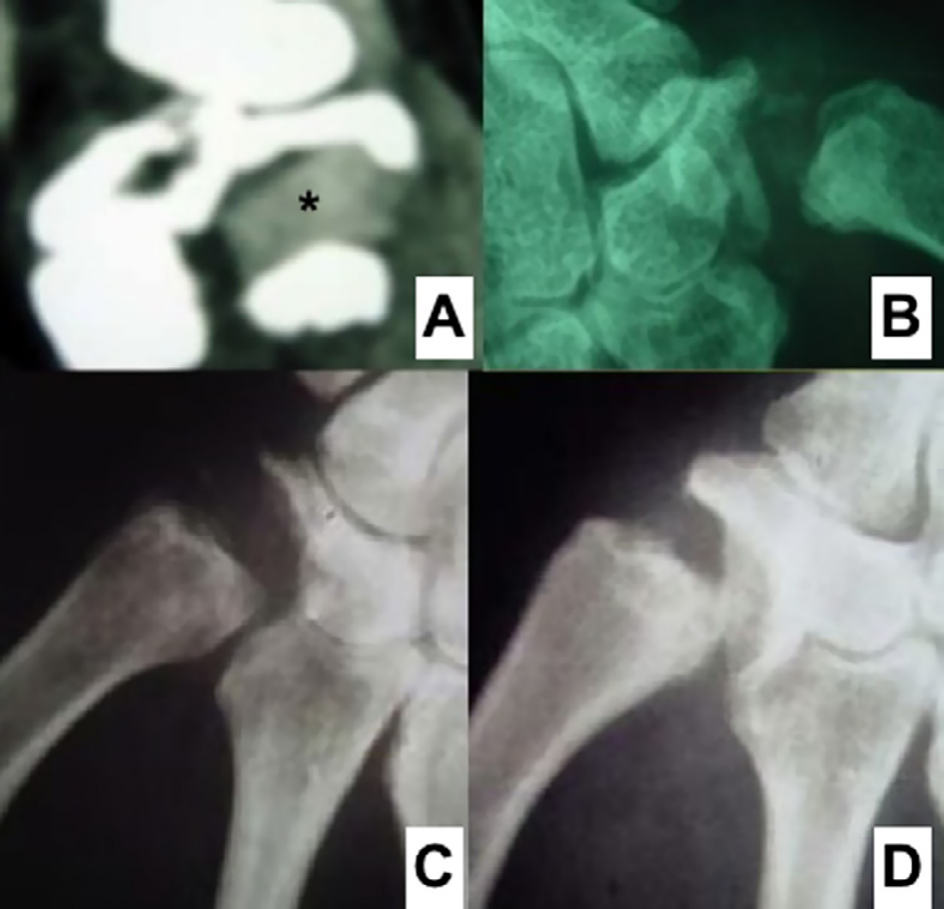
CT scan aspect of a graft (*) in the space created by trapeziectomy (A), slight metaplasia appeared sometimes with time without any clinical consequences (B), Narrowing of the space appeared with high follow-up but less than trapeziectomy alone (C & D).

Tropet et al. [36] reviewed 97 patients (116 thumbs) operated for basal joint arthritis between 1992 and 2005, 90% of them by the same surgeon. These patients were reviewed with a mean follow-up of six years. Ten patients were lost to follow-up. Five grafts had to be removed and there was one case of complex regional pain syndrome. As a consequence, 100 thumbs were available for review and analysis. Three complications during graft harvesting were noted. At the latest postoperative follow-up, 83% of patients were pain free and 96% of patients were satisfied, with a DASH score of 18.7 (range 0–56.7). The implanted graft was evaluated by X-rays, CT scan, MRI (in 19 cases), and histology (in 10 cases). The graft was viable at the follow-up. There was partial ossification in the graft and the scaphometacarpal space was narrower.

#### 5.1.1 Change in graft appearance over time

Nineteen MRIs were performed in patients at more than 5 years of postoperative follow-up [36]. The T1-weighted images provided the best assessment of graft viability. The graft had an isointense signal, similar to muscle. This likely meant that the graft was vascularized; otherwise it would have a hypointense signal. The fibrovascularized buds colonizing the cartilage graft probably played a role in this signal. The graft did not have a bone-type hyperintense signal. Therefore, it had not completely become bone, as certain plain X-rays may suggest. In T2-weighted images, the graft systematically appeared as a hypointense signal. This signal was suggestive of graft necrosis, whereas the T1 images suggested the opposite. How did these MRI signals correlate to the histological changes in the graft? Histological analysis found a certain degree of osseous metaplasia in the graft. This bone formation within the graft creates an artifact, which explains the signal contradiction between the two types of MRI sequences. On these 19 MRI scans, there were 14 instances of one or several hyperintense signals in the areas surrounding the graft, but also 16 within the graft, which corresponded to osseous metaplasia (Figure 4).

#### 5.1.2 Graft wear, viability, and ossification

In the patients who underwent MRI at different follow-up times, no graft wear was noted, but it was very difficult to compare the MRI images taken over time. In patients where sagittal views were taken, there was evidence of the graft molding itself into the base of the first metacarpal.

Histological analysis of ten grafts was carried out between two and 46 months postoperative, when the joint dislocated or after revision surgery. Neovascularization of the cartilage graft was noted, but there were areas of chondrocyte necrosis with fibrous tissue development and osseous metaplasia.

Ossification within the graft corresponding to osseous metaplasia was probably related to the presence of fibrovascular granulation tissue formation. This ossification varied greatly from patient to patient. It appeared either at the center or periphery of the graft. On X-rays, it can take on a trapezoid appearance; at the latest follow-up, the X-rays may give the impression of a nearly completely rebuilt trapezium.

#### 5.1.3 Minimal narrowing of the scaphometacarpal space

The scaphometacarpal space had narrowed to 2 mm. This was not related to any clinical outcomes in the multivariate study. It was most apparent during the first year and corresponded to the graft’s adaptation to the base of the first metacarpal. Graft lysis was found in four out of 100 cases. This lysis was present every time the trapezium had been completely excised. For this reason, Tropet deems it essential to retain a part of the trapezium when treating basal joint arthritis.

### 5.2 Treatment of radiocarpal osteoarthritis

In cases of radiocarpal osteoarthritis [21], a rare post-traumatic or degenerative condition, fusion should be avoided. This condition is most often due to localized radiocarpal impaction in a young, highly active patient with high expectations. The osteocartilaginous defect must be fully evaluated preoperatively using thin-slice CT; the only limitation for this indication is the amount of bone and cartilage loss. The preferred surgical approach is on the damaged side, which can also be determined based on the preoperative CT. The graft is harvested so as to position the bone side against the radius. The cartilage side is trimmed as needed and placed against the radiocarpal joint.

Seven patients were operated using this technique. They all presented substantial wrist pain and stiffness initially. Their mean age was 49.2 years (range 22–72 years). They were reviewed at a mean follow-up of 22.5 months (range 6–38 months). Both clinical and imaging evaluations were performed. The mean Herzberg score [13] was 72/100 (range 54–82). The postoperative DASH score was 30.3 (range 22.5–51.7). Two biopsies were taken during material removal; both showed viable grafts. One failure was observed: this was a technical error where the size of the radius lesion was underestimated in a female patient with radiocarpal osteoarthritis that was very likely secondary to scaphoid sepsis. Substantial lysis of the proximal pole of the scaphoid with osteocartilaginous lesions was observed in the radius.

### 5.3 Treatment of periscaphoid osteoarthritis (SNAC & SLAC)

In cases of periscaphoid osteoarthritis, wrist pain and weakness [17, 31] continue to plague patients. There is no clear-cut gold standard treatment, but proximal row carpectomy and intracarpal fusion are the two main types of procedures carried out. To our knowledge, there have not been any randomized comparative studies performed up to now. The primary goal is to reduce pain as much as possible while maintaining function in very active patients. These patients can be quite vocal and their relationship with pain and activity can be very childish: they often push themselves to the utmost of their ability and too often end up in surgery with an extremely painful clinical picture. In these patients, reduced mobility is often a true problem, particularly in radial/ulnar deviations.

In this disease condition, whether nonunion or a scapholunate ligament lesion coexists with osteoarthritis, the proximal pole of the scaphoid is resected. This is logical in cases of scapholunate advanced collapse (SLAC) because this part of the scaphoid is arthritic. In scaphoid nonunion advanced collapse (SNAC), this approach has been criticized because the middle and distal part of the scaphoid are arthritic. It is critical that less than the proximal two-thirds of the scaphoid be resected and the osteochondral cartilage graft (bone part toward the scaphoid and cartilaginous part toward the radius) be attached with at least two pins. We had not used screw fixation with this graft until very recently [22]. Now that 2-mm screws are available, we have modified our technique. And because of the technical difficulty of placing an osteochondral graft, on some occasions we have implanted a graft perpendicularly without taking into account whether it was bone or cartilage. Eighteen patients were operated over a 6-year-period using this technique: 16 were men with a mean age of 47.7 years (range 26–62 years). There were 12 SNAC cases and six SLAC cases. The mean follow-up was 4.1 years (range 6 months to 9.5 years). Graft union was observed in 17 patients at 3 months, without graft resorption. Although there is a potential for osteoarthritis to progress within the carpus, this had not occurred in our patients at the latest follow-up. The subjective evaluation found 17 patients who were satisfied or very satisfied, with improvements observed relative to the preoperative condition. Most specifically, the O’Brien functional [10] score showed five excellent results, ten good results, and three fair results.

### 5.4 Kienböck’s disease

Advanced Kienböck’s disease is treated by proximal row resection or by partial arthrodesis. Between 2007 and 2009 four patients of mean age 40 years (32–51) were operated using replacement of the lunate with a free costochondral autograft [14]. Like cartilage graft in basal thumb arthritis, lunate is resected dorsally and cartilage graft is placed “free” in the cavity created. With a mean follow-up of 27 months (6–36) results show the disappearance of pain at rest and during daily activities for all patients and a mean DASH of six. Flexion-extension was 108% of controlateral side and grip strength 83% compared to the opposite side. Radiological evaluation showed no disease evolution. No complication was reported. Functional improvement was significant with good results compared to conventional techniques. Alternative techniques have been proposed for the replacement of the lunate, each with its specific problems. Lunate replacement by a costochondral graft is possible because studies showed vitality of this free graft up to five years. It also allows subsequent surgery. The absence of carpal collapse and good functional results are encouraging but the follow-up is short. A long-term study is needed to confirm findings.

## 6 Conclusion

We wanted to take the mystery out of harvesting a rib cartilage graft, as this type of graft can be used in multiple ways in upper limb surgery. When used for reconstruction procedures, it can be easily and reliably matched to the defect size and can act as a free spacer or fixed graft. Partial and total fusions are traditional solutions for cases of degenerative disease in the hand and the wrist. Functional results after fusion are predictable in painful wrists having undergone multiple surgeries. We believe that pain can be eliminated with rib cartilage grafting while preserving whatever mobility the arthritic patient still has. Although there are alternatives to which grafting should be compared for treatment of basal joint arthritis and periscaphoid osteoarthritis, this option provides true resurfacing in cases of joint malunion. The absence of other conservative solutions to reconstruct a destroyed joint surface is the main argument in favor of this surgical technique, which we are reporting here for the first time.

## Disclosure and conflict of interest

L. Obert is a consultant for FX Solution, Zimmer, SBI, Synthes & Depuy, Medartis, Evolutis, Biotech & wright, Argo.

Every other author certifies that he has no commercial associations that might pose a conflict of interest in connection with the submitted article.

Each author has no conflict of interest in connection with this article.
